# Supplementary material for: A Novel Physical Mobility Task to Assess Freezers in Parkinson’s Disease
Source: Healthcare (Basel). 2023 Jan 31;11(3):409. doi: 10.3390/healthcare11030409 (PMC9914147; doi:10.3390/healthcare11030409)
Supplement: Supplementary file 1 [file healthcare-11-00409-s001.zip › supplementary tables.pdf]

## DUAL TASK RESULTS

Table S1 presents how many times each number was mentioned in the audio track. For each trial, a digit from the set of 1 to 9 was drawn without replacement.

Audio transcript: 459616619359126578814596166

Table S1. How many times each digit from 1 to 9 is mentioned in the audio track.

| Number 1 | Number 2 | Number 3 | Number 4 | Number 5 | Number 6 | Number 7 | Number 8 | Number 9 |
|---|---|---|---|---|---|---|---|---|
| 5 times | once | once | twice | 5 times | 7 times | once | twice | 4 times |

Table S2 shows the volunteer's answer during the DMT for each trial of the dual task. The table is colored in green and red to show the right (green) and wrong (red) answers given by the volunteers. The second column shows the percentage of mistakes per volunteer, showing that only one participant did not make a mistake during the dual task. The percentage mean of the volunteers' mistakes is 45.65%.

Table S2. Answers of the volunteers during the Digit Monitoring Task (DMT) for each trial of the dual task. Correct answers in green and wrong answers in red.

| GFOG+ | % OF MISTAKES | OFF | | | | | | ON | | | | | |
|---|---|---|---|---|---|---|---|---|---|---|---|---|---|
| VOLUNTEER | | DMT1 | R1 | DMT2 | R2 | DMT3 | R3 | DMT1 | R1 | DMT2 | R2 | DMT3 | R3 |
| 1 | 50% | 3 | 1 | 6 | 7 | 8 | 3 | 9 | 4 | 4 | 1 | 5 | 4 |
| 2 | 0% | 6 | 7 | 8 | 2 | 7 | 1 | 1 | 5 | 4 | 2 | 3 | 1 |
| 3 | 83.3% | 5 | 3 | 2 | 2 | 4 | 1 | 7 | 1 | 3 | 2 | 6 | 6 |
| 4 | 33.3% | 7 | 0 | 9 | 4 | 6 | 5 | 3 | 1 | 2 | 1 | 8 | 2 |
| 5 | 50% | 1 | 4 | 7 | 1 | 8 | 1 | 4 | 2 | 9 | 2 | 2 | 1 |
| 6 | 33.3% | 2 | 0 | 9 | 4 | 1 | 5 | 6 | 7 | 7 | 1 | 5 | 4 |

| | | | | | | | | | | | | | |
|---|---|---|---|---|---|---|---|---|---|---|---|---|---|
| 7 | 50% | **4** | **2** | **3** | **2** | **6** | **6** | **5** | **4** | **9** | **4** | **2** | **1** |
| 8 | 50% | **9** | **4** | **7** | **1** | **3** | **0** | **2** | **1** | **6** | **3** | **5** | **4** |
| 9 | 33.3% | **2** | **1** | **4** | **2** | **9** | **4** | **5** | **4** | **3** | **2** | **8** | **2** |
| 10 | 83.3% | **2** | **4** | **1** | **7** | **8** | **7** | **4** | **2** | **3** | **2** | **6** | **6** |
